# Pupil response patterns distinguish true from false memories

**DOI:** 10.1038/s41598-023-44362-6

**Published:** 2023-10-11

**Authors:** Alex Kafkas, Travorn Brown, Nifemi Olusola, Chaodong Guo

**Affiliations:** School of Health Sciences, Division of Psychology, Communication and Human Neuroscience, University of Manchester, Oxford Road, Manchester, M13 9PL UK

**Keywords:** Psychology, Learning and memory

## Abstract

Memory is reconstructive and error-prone, which make memory illusions very common in everyday life. However, studying memory illusions can provide valuable insights into how memory works. Pupil response has emerged, in recent years, as an indicator of memory encoding and retrieval, however its validity as a measure of memory success is debated. In this study, we explored whether pupil response patterns can differentiate true from false memories and whether variations in the temporal dynamics of pupil response can elucidate the mechanisms underlying false memory creation. The Deese-Roediger-McDermott (DRM) paradigm was employed to generate false memories in two separate experiments involving visual and auditory stimuli. Pupil responses effectively differentiated true from false memories based on variations in pupil amplitude at different temporal components. This discrimination remained consistent across both experiments, with slightly stronger effects in the auditory condition, aligning with the more pronounced false memory effects in this condition. Notably, differential pupil responses between true and false memories varied based on the type of memory involved at recognition. These findings provide valuable insights into the cognitive processes underlying memory distortions, with implications for theoretical frameworks and real-world contexts.

## Introduction

Memory distortions and illusions are extremely common in everyday life, signifying the malleability of memory retrieval. These may include false memories for events that have never been encountered before, or they may involve distorted memory content for truly encountered material^1^. In either case, false memories can feel as real and strong as truly experienced events, which testifies to the reconstructive nature of memory^2^. The implications of the imperfectness of the memory system are immense and encompass multiple facets of human life, including legal, interpersonal, clinical practice and social settings^3^. At the same time, memory distortions allow scientists to gain a better understanding of memory function and mechanisms, the same way visual illusions provide insights about visual perception mechanisms^4^. In the present study, we sought to explore whether pupil response, which has emerged as a measure of memory formation, memory strength and retrieval quality^5–8^, discriminates true from false memories.

### Pupil response and long-term memory

Changes in pupil amplitude have been shown to predict the strength and the type of subsequent memory^6,8–11^. The pupil response also discriminates between the types of encoding mechanisms engaged when different types of novelty are detected^8^. At retrieval, different memory outcomes result in different pupil patterns, with accurately recognised old items associated with greater pupil dilation than accurately discriminated new stimuli – i.e., the pupil old/new effect^5,7,12^. The pupil response also discriminates between the different kinds of memory that support recognition memory decisions^5,13^. Specifically, according to a prevalent theory of recognition memory, familiarity denotes recognition of the item itself as a previously presented occurrence, while recollection involves bringing to mind associative details from the encoding episode^5,14–17^. Consistent with the pupil old/new effect, new responses have been found to elicit the smallest degree of pupil dilation, familiarity responses to elicit intermediate levels of dilation, while recollections involved the highest level of pupil dilation^5^. This means that pupil response does not simply reflect memory strength, but more importantly it indicates successful retrieval of associative elements from an episode^18^.These previous findings highlight the sensitivity of the pupil response in reflecting memory formation and retrieval. However, whether it has the capacity to discriminate between true and false memories is still to a great extent understudied and debated e.g.,^12,19^.

Preliminary evidence comes from studies using the recognition memory paradigm, in which accurate (i.e., hits and correct rejections) and inaccurate (i.e., misses and false alarms) old/new responses can be discriminated. For example, increased pupil dilation for false alarms (new stimuli classified as old) than correct rejections (new stimuli accurately classified as new) has been reported^13,20^, which indicates pupil sensitivity to subjective recognition of stimuli as old or new. On the other hand, pupillary effects have previously been reported in the case of amnesic patients^21^, who showed a pupil old/new effect in a recognition memory task despite their poor behavioural performance. More recently, Kafkas and Montaldi^12^ found that pupil timeseries (pupil responses across time) discriminated true from false old/new decisions, with earlier pupil amplitudes reflecting the true, old or new, status of stimuli and later pupil amplitudes reflecting the subjectively reported old or new status, which in the case of misses and false alarms was erroneous. Contrary to these findings, other studies, also employing recognition memory paradigms, have failed to provide evidence for the capacity of pupil response to discriminate veridical from false memories^19,22^. Considering the sensitivity of the pupil response to various perceptual, visual and cognitive factors, methodological differences between these studies may well explain the disparity. However, to date no study has explored this question using paradigms specifically designed to separate true from false memories.

Taken together, despite the reconciliation shown in Kafkas and Montaldi study^12^, in relation to the sensitivity of pupil response to accurate and inaccurate old/new responses at different timepoints of recognition processing, it remains more or less unresolved whether the pupil response, as a measure of episodic memory, can discriminate veridical from false memories. To determine whether the pupil response is a reliable indicator of long-term memory retrieval and not just the output of other factors – e.g., working memory load, arousal or response preparation^23–26^, it is relevant to examine how well it can distinguish true from false memories in paradigms designed to target and generate false memories.

### False memory and the DRM paradigm

One of the most widely studied paradigms for inducing memory distortions is the Deese-Roediger-McDermott (DRM) paradigm^27,28^. In this paradigm, participants study lists of words that are semantically related to a non-presented critical lure word. For example, participants may study words such as *apple, vegetable, orange, citrus* etc., which are all related to the critical lure *fruit*. Later on, participants are asked to recall or recognise the studied words and the critical lures. Typically, participants show high rates of false recall and recognition for the critical lures that were never presented but were highly associated with the studied words^29–31^. This phenomenon demonstrates how memory retrieval can be influenced by prior knowledge, top-down processing and expectations based on semantic associations.

Various theoretical models have been proposed to account for false memories in the DRM paradigm, which can be summarised within two broad frameworks, the activation-monitoring framework e.g.,^4,32^ and the fuzzy-trace theory e.g.,^33–35^. Briefly, according to the activation-monitoring framework, false memories in the DRM paradigm are generated due to the *activation* of associated or similar items which are organised closer together in pre-existing semantic representations. When a word is encountered, either at encoding or at retrieval associative activation spreads to semantically similar concepts, making them more likely to be falsely retrieved at test^36–39^. A source-monitoring attributional component^40^may, nevertheless, diminish the effect of spreading activation and therefore can reduce the generation of false memories. In contrast, according to the fuzzy-trace theoretical model, false memories in the DRM paradigm stem from two types of memory traces produced when exposed to an event or stimulus. The verbatim trace reflects detailed or surface-related memory of a previously encountered stimulus and may allow rejecting a false memory. On the other hand, the gist trace comprises semantic relations and meaning and is responsible for false memories generated at test^33^. The extraction of gist elements from a study episode makes memory illusions more likely, especially when the verbatim traces are not detailed. In contrast, recalling verbatim elements allows false memory rejection^41^.

These theories differ in their emphasis on the roles of encoding processes (e.g., gist extraction vs. item-specific processing), retrieval processes (e.g., activation vs. monitoring and/or decision-making), and memory representations (e.g., verbatim vs. gist traces vs. feature vectors) in explaining memory distortions. However, they also share important commonalities, such as the influence of semantic associations (or gist coherence) and contextual cues on memory performance. These mechanisms are relevant to consider when interpreting systematic variations in measures associated with false memory.

### The present study

In the present study, we systematically explored whether the pupil response can discriminate true from false memories using a well-established false memory paradigm. The DRM paradigm was utilised in two forms, one visual (printed words) and one auditory (spoken words), to explore the degree to which the pupillary response at test can discriminate true from false memories. This investigation will provide valuable evidence as to whether the pupil response is a valid measure of memory retrieval. Secondly, the temporal dynamics of the pupil effects, during memory processing, may allow a consideration of the mechanisms involved in the generation of false memories, especially when different types of memory experience are involved.

At least three hypothetical scenarios can be described in relation to the ability of the pupil response to discriminate true from false memories. The first possibility is that the pupil response only reflects subjective memory decisions and therefore the old/new effect would accompany hits but also falsely recognised stimuli without discriminating between the two. i = In this case, irrespective of the actual old/new status of the stimuli an old response (to either truly old or new stimuli) will result in increased pupil amplitude. Another possibility is that the pupil response exclusively reflects the old/new status of the stimuli irrespective of subjective memory response. In this case, the pupil response will be predominantly driven by true, but not false memories. The final possibility is that a combination of both objective and subjective elements of memory are reflected on the pupil responses and therefore the pupil amplitude at retrieval will be modulated by both true and false memories.

The present study also investigated whether the pupil response patterns that may separate true from false memories, vary depending on whether the memories are based on familiarity or recollection. To this end, the kind of memory was also taken into account in a recognition memory task by employing a modified remember/know procedure to probe memory experience on a trial-by-trial basis^16,42–44^.

## Methods

### Participants

In total, data from 80 participants are reported here across two experiments (40 in each experiment). Forty-five participants in Experiment 1 and a different group of 44 participants in Experiment 2, gave informed consent and participated in the studies in exchange for course credits. Data collection was in accordance with the University of Manchester regulations for studies with human participants and the experiments were approved by the University of Manchester Research Ethics Committees. Data from 5 participants in Experiment 1 and 4 participants in Experiment 2 were excluded due to incomplete or noisy eye tracking recordings (see Data Analyses section for criteria). The remaining 40 participants (35 female) in Experiment 1 had a mean age of 19.8 years (SD = 1.20), while the 40 participants in Experiment 2 had a mean age of 19.3 years (SD = 1.11). The sample size in the two experiments was consistent with previous memory and pupillometry studies^8,12^, while a power analysis using the GPower tool^45^ (parameters: eta-squared = 0.11, effect size f = 0.35; power = 0.99) indicated that 40 participants strongly replicates the previously reported pupil old/new effect from Kafkas and Montaldi^12^. All participants were native English speakers, with normal or corrected-to-normal (with contact lenses) vision and self-reported no history of psychiatric or neurological disorders. They were asked to abstain from caffeine and alcohol consumption 24 h before participating in the study. No participant reported altered energy or concertation due to caffeine withdrawal at the end of the experiment.

### Materials and design

The two experiments used similar design and procedures with the main difference being the delivery of visually presented words in Experiment 1 and auditorily presented words in Experiment 2. In both experiments, the 24 lists of words reported in Roediger & McDermott^28^ were used. Each list consists of a list of 15 words, which are all semantically associated with one critical lure or target word. For example, the words *thread, pin, eye, sewing, sharp, point, prick, thimble, haystack, thorn, hurt, injection, syringe, cloth and knitting* are semantic associates, of progressive association strength, to the critical word *needle.* Within each experiment, a within-subject design was implemented including an encoding, a filled interval, and a recognition memory task. As in the original DRM paradigm (Experiment 2 in Roediger and McDermott, 1995), the 24-word lists were divided into 3 groups of 8 lists each for counterbalancing purposes. Participants encoded 16 lists, randomly selected from the 3 groups of lists, and words from the remaining 8 lists were used as new foils in the recognition memory task. The filler task included an unrelated verbal and an arithmetic task with a total duration of 10 min. In the recognition block, a total of 96 words were presented, consisted of 48 studied at encoding and 48 new items. The 48 studied items comprised 3 items selected from each of the 16 studied lists. Following Roediger & McDermott^28^, from each list, items in the serial position 1, 8 and 10 were selected to be shown again at recognition. The 48 unstudied items comprised 24 words derived from the 8 unstudied lists (again from serial positions 1, 8 and 10), 16 critical lures from the studied lists and 8 critical lures from the unstudied lists.

### Procedure

**Figure 1:**
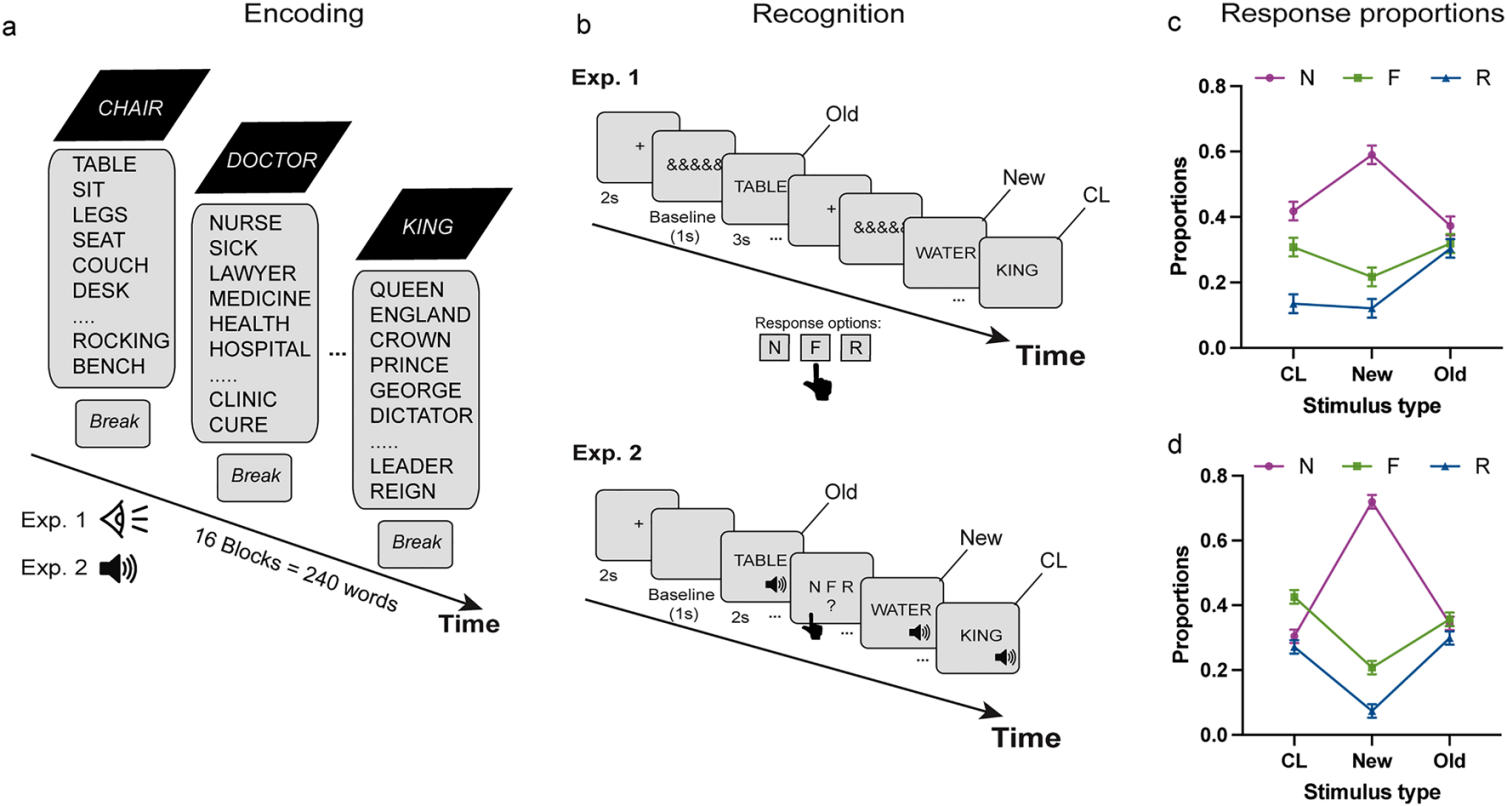
Design of Experiments 1 (visual) and 2 (auditory) and behavioural response proportions in the two experiments. (**a**) At encoding, participants studied 16 lists of words (15 words per list) by reading each word aloud (Experiment 1) or by listening to each word twice in each trial (Experiment 2); (**b**) In the recognition task, studied (old), unstudied (new) and critical lures (CL) were presented and participants were asked to take recognition memory decisions using N = new, F = familiar, R = recollected response options; (**c**) Proportion of N, F, R responses across the three types of stimuli in Experiment 1 (**c**) and Experiment 2 (**d**). Standard errors show ± 1 standard error of the mean.

Each experimental session started with the encoding task, followed by the filled interval and then the recognition memory task during which the eye tracking data were recorded. At encoding participants were presented with 16 lists of 15-words each, giving a total of 240 trials (see Fig. 1). In Experiment 1, each word was presented for 2 s after a fixation point (1 s) and participants were asked to read each word aloud. In Experiment 2, each trial was delivered auditorily within a 2-s window, during which each word was played twice. Prior piloting showed that playing each word twice at encoding resulted in comparable memory performance as when visual presentation was used. Within each word list the words were presented sequentially and their order was held constant across participants, as standard in the DRM paradigm^28^. After each list of 15 words participants were given the chance to take a short self-paced break before proceeding to the next list. The average break duration was 1992 ms (SD = 875) in Experiment 1 and 2022 ms (SD = 883) in Experiment 2. A practice block of three words, not presented anywhere else in the main study, were used before the encoding task. A filed interval of 10 min (constant across participants) followed the encoding block during which participants were asked to perform unrelated verbal and arithmetic tasks.

After the filler task, participants were trained on the recognition memory instructions. They were instructed that studied and unstudied words will be presented and that they will be given the options to report studied words as familiar or recollected, whilst unstudied words could be reported as new. A familiar response meant that the word could be recognised as previously presented one, without bringing to mind any additional contextual details from the time of encoding. On the other hand, a recollection response meant that a word was recognised as previously studied on the basis of retrieved contextual detail from encoding (e.g., a thought generated at encoding related to the specific stimulus). Participants were instructed not to use the familiar/recollected options as indicators of confidence, but rather as indicating whether cued recall of additional details from the encoding episode was present (recollection) or not (familiarity)^8,42,46^. Prior commencing the recognition task, participants had the chance to ask questions, they explained the response options to the experimenter to ensure understanding and they practiced the response options on 5 words (3 from encoding practice).

Before starting this task, each participant’s head was stabilised using a chinrest and a nine-point calibration of the eye tracker was performed. Eye tracking data were collected from this block, in both experiments, while participants engaged in recognition memory decisions for the 96 words. A standard computer keyboard was used for collecting behavioural responses. In Experiment 1, each word appeared after a fixation cross (2 s) and a baseline period (1 s) which presented a sequence of 8 ampersand symbols (&&&&&&&&&&). Pupil data recorded during this period were used as the trial-specific baseline traces (see below). Each word was presented for a maximum of 3 s during which a recognition decision was required (R, F, N) while the stimulus was on the screen (see Fig. 1b). In Experiment 2, each word was delivered auditorily during a 2-s window, while a grey blank screen was presented, followed by a response screen (3 s) during which participants were asked to provide a recognition memory response (R, F, N). Each word was played after a fixation cross (2 s) and a baseline period (1 s), during which a blank grey screen was visible—the same as during word delivery. In both experiments, participants were told that their eye movements would be recorded, while they process (read or hear, respectively for Experiment 1 and 2) word stimuli with no reference to false memories or pupillometry. Participants were debriefed at the end of the experiment in relation to the aims of the study.

### Apparatus, eye tracking, pre-processing and data analysis

A quiet and moderately lit (at 250 lx) testing room was used for participant testing. A computer with a 19-inch monitor (1280 × 1024 resolution) and a standard keyboard were used in both experiments. In Experiment 2, noise cancelling headphones were used to deliver the auditory stimuli at maximum volume. Participants retained a distance of about 70 cm from the computer screen (and the eye tracking camera) throughout the experiment and a chinrest was used to stabilise participants’ head during the eye tracking recording period (recognition block).

Pupil diameter from participants’ left eye was recorded with an ASL (Applied Science Laboratories, Model Eye-Trac 6000; 60 Hz) infrared eye tracking system. A standard nine-point calibration was performed just before each participant started the recognition memory task. Pupil diameter data recordings were synchronised to start at the beginning of the trial-specific baseline and lasted throughout the presentation of each stimulus (i.e., − 1000 ms to 3000 ms from stimulus onset in Experiment 1 and -1000 ms to 2000 ms from stimulus onset in Experiment 2). These trial-specific eye tracking data were stored for later off-line analysis.

Raw pupil dimeter data were analysed using established pupillometry protocols see e.g.,^8,12^. Specifically, blinks and partial closures of the eyelid were automatically identified during recording and were discarded. To identify residual artefacts in the recordings the grand mean of pupil recordings within each trial was calculated and those pupil recordings diverging by more than three standard deviations from the grand mean were discarded. These pupil traces are residual artefacts normally preceding or following partial eyelid closures and blinks. Trials with more than 50% discarded pupil traces were excluded from the analyses, while participants with 40% or more excluded trials were excluded from the final sample. In total, across the two experiments, 9 participants were excluded from the analysis due to incomplete pupil data. Discarded pupil recordings in the final sample were less than 6% of the total recorded traces (across both experiments) and therefore linear interpolation was not used to replace missing recordings.

Pupil timeseries were analysed and are reported in the present study. In Experiment 1, pupil size in each trial was recorded for the period of stimulus presentation up to the point of behavioural response (variable duration) as well as during the preceding baseline period (constant duration of 1 s). In Experiment 2, pupil size in each trial was recorded for the entire 2-s stimulus presentation window as well as during the preceding baseline period (1 s). Each pupil recording was subtracted from the average trial-specific baseline pupil size and therefore was expressed as deviation from a zero-point baseline. Baseline-corrected pupil responses were standardised to a length of 10 time-points, from stimulus onset to the point of behavioural response (Experiment 1) or until the end of the 2-s period (Experiment 2).

Linear mixed effects analyses with maximum likelihood estimation were conducted to explore the effect of the fixed factors, stimulus type (old, new, critical lure) and memory type (familiar, recollected, new), on the behavioural (proportions and RTs) and pupil response measures (see Table S2 in Supplementary Tables for model equations and model fit parameters) . Proportions represent the proportion of each response type (F, R, N) divided by the maximum number of old (48), new (32) or CL (16) stimuli in the recognition block. As calculating proportions requires averaging across multiple trials, this analysis contained 9 scores for each participant corresponding to all combinations of reported memory type (N, F, R) and stimulus type (old, new, CL). This analysis allowed to determine the probability of each response type (F, R, N) across the three types of stimuli. Especially for the CL stimuli, these outcomes indicate how likely were these stimuli to generate false memories as compared to new and old stimuli. Furthermore, memory performance was also calculated as the hit rate – false alarm rate to identify whether overall discrimination of old and new stimuli was above chance.

Analyses of RTs are reported for Experiment 1, but not for Experiment 2, as recognition memory responses in the latter experiment were required after the auditory delivery of the stimuli, which renders them less informative. Time at recognition (with 10 timebins) was specified as a fixed factor in the mixed model analysing pupil response patterns. Pupil data were not averaged across trials in this model and therefore each trial was specified in the model and contained pupil responses across 10 timebins. Participants were specified as a random effect (random intercept) in all the models reported here. A model including words/trials as a random effect, to control for any differential effect of word items on the pupil response, did not change the patterns of results and therefore the simpler model is reported here. This indicates that the differential pupil patterns were mainly driven by the fixed factors of interest, while variation across words played minimal role. Type III Wald F tests are reported in all analyses, with Satterthwaite’s method for degrees of freedom approximation^47,48^. A standard alpha (*p* < 0.05) was adopted for all analyses while Bonferroni-Holm corrected *ps*^49^ are reported for post-hoc pairwise comparisons.

## Results

### Behavioural effects

Mean proportions of familiar, recollected and new responses and their RTs across the three types of stimuli at recognition (old, new, CL) are presented in Fig. 1c,d and in Table S1 (Supplementary Tables; RTs for Experiment 1 also presented in the table). Overall, recognition memory performance was significantly different from chance in both experiments, collapsed (Experiment 1: M = 0.28, SD = 0.39, *t*_39_ = 4.66, *p* < 0.001; Experiment 2: M = 0.37, SD = 0.27, *t*_39_ = 8.69, *p* < 0.001) and separately for F (Experiment 1: M = 0.10, SD = 0.22; *t*_39_ = 2.87, *p* = 0.007; Experiment 2: M = 0.15, SD = 0.18; *t*_39_ = 5.17, *p* < 0.001) and R responses (Experiment 1: M = 0.18, SD = 0.25; *t*_39_ = 4.71, *p* < 0.001; Experiment 2: M = 0.22, SD = 0.18; *t*_39_ = 7.75, *p* < 0.001), while R performance was slightly higher than F performance (Experiment 1: *t*_39_ = 1.96, *p* = 0.058; Experiment 2: *t*_39_ = 1.93, *p* = 0.061). These findings indicate that despite the false memory manipulation, the participants were able to successfully discriminate truly old from new stimuli.

To explore the distribution of F, R and N responses across the three types of stimuli, a linear mixed model with stimulus type (old, new, CL) and memory type (F, R, N) was conducted on the response proportion rates. Proportions were equally distributed across the three types of stimuli (Experiment 1: *F*_2, 360_ = 1.86, *p* = 0.16; Experiment 2: *F*_2, 360_ < 1), but they differed across the memory outcomes (Experiment 1: *F*_2, 360_ = 71.75, *p* < 0.001; Experiment 2: *F*_2, 360_ = 101.1, *p* < 0.001), with higher proportion of N vs F vs R (N > F > R; all *p*s < 0.001). The significant interactions indicated differential proportion of F, R and N responses across the three types of stimuli (Experiment 1: *F*_4, 360_ = 15.61, *p* < 0.001; Experiment 2: *F*_4, 360_ = 92.4, *p* < 0.001). Specifically, in Experiment 1 (Fig. 1c), post-hoc tests revealed that familiarity responses were equally likely for CL and old stimuli (*t*_360_ < 1), while new responses were disproportionally more for new stimuli relative to either CL or old stimuli (all *p*s < 0.001). Finally, recollections were more likely for old than either new or CL stimuli (all *p*s < 0.001). In Experiment 2 (Fig. 1d), familiarity responses were more likely for CL and old stimuli relative to new ones (all *p*s < 0.001), while new responses were significantly higher in the case of new stimuli relative to either CL or old stimuli (all *p*s < 0.001). Finally, recollections were higher for CL and old stimuli than new stimuli (all *p*s < 0.001), while they did not differ between CL and old stimuli (*t*_360_ < 1). These findings confirm that CL stimuli were more likely to generate false memories than unrelated new foils. False memories tended to rely more on familiarity than recollections in Experiment 1 (visual task), while in Experiment 2 (auditory task) false memories to CL stimuli resulted in increased familiarity and recollection responses.

The analysis on RTs from Experiment 1 indicated matched RTs across old, new and CL stimuli (F_2, 290_ = 1.17, *p* = 0.31). However, RTs significantly differed across the three memory types (*F*_2, 290_ = 22.84, *p* < 0.001) with faster RTs for new and R responses relative to F ones (all *p*s < 0.001). Finally, the significant stimulus type by memory type interaction (*F*_2, 290_ = 3.74, *p* = 0.006) indicated faster N responses to new than to old or CL stimuli (all *p*s < 0.01), while faster R responses were the case for CL and old than new stimuli (all *p*s < 0.01). These effects suggest sufficient false memory generation for CL stimuli.

### Pupil effects

#### Experiment 1

**Figure 2:**
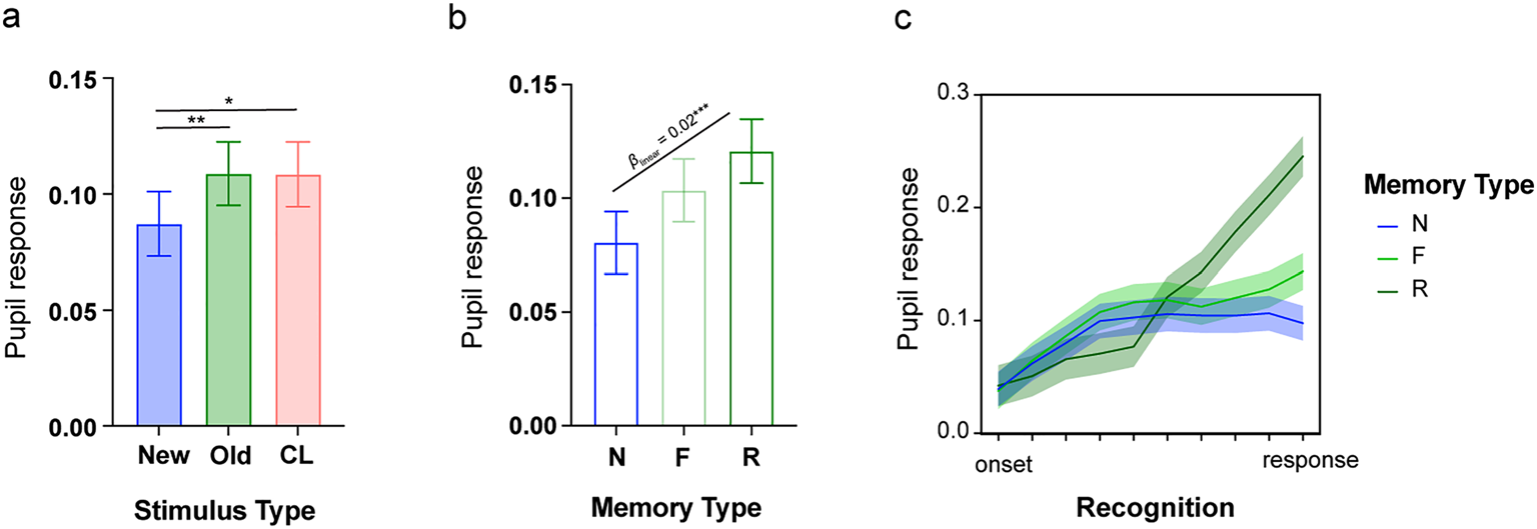
Pupil response across the different stimulus types (new, old and CL; a) and across reported memory outcomes (N = new, F = familiar, R = recollected; b and c) in Experiment 1. Error bars (**a**, **b**) and shaded areas (**c**) show the standard error of the mean. * *p* < 0.05; ** *p* < 0.01; *** *p* < 0.001.

**Figure 3:**
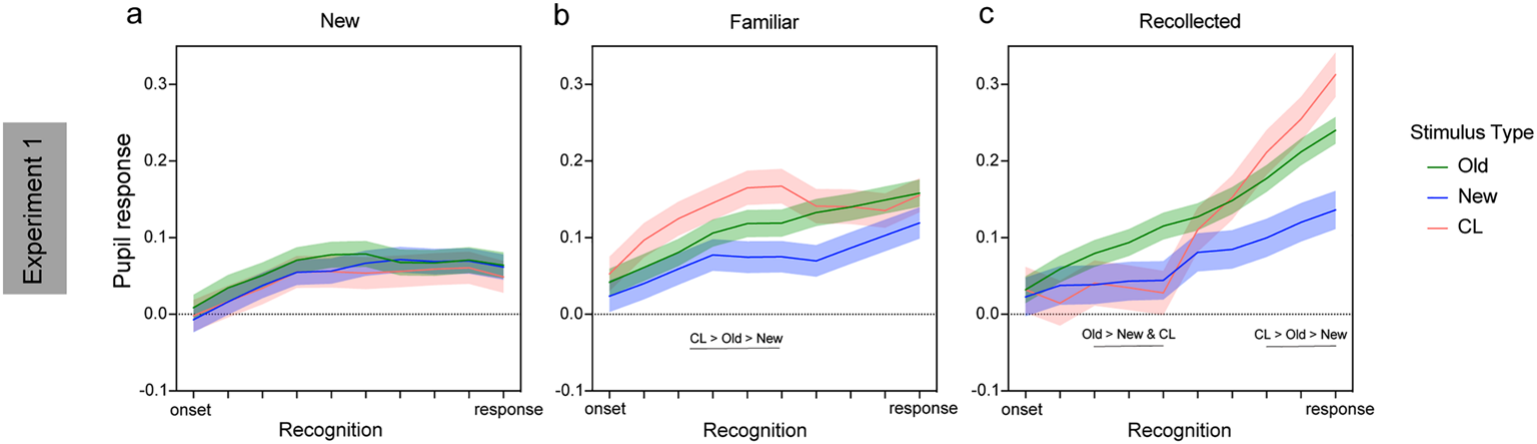
Pupil response timeseries during recognition for the different stimuli (old, new, CL) and reported memory – (**a**) new; (**b**) familiar (**c**) recollected in Experiment 1. Shaded areas on the timeseries show the standard error of the mean. Lines indicate significant effects at *p* < 0.05.

In Experiment 1, the analysis was conducted on a total of 3815 trials across participants, separated as 838 recollections, 1149 familiarity and 1828 new responses across old, new and CL stimuli. The linear mixed effects analysis on pupil response was conducted with fixed factors: stimulus type (old, new, CL), reported memory type (F, R, N) and time (10 time-bins) and the random effect of participants (intercept). This analysis showed significant differences in pupil response across the different stimulus types (*F*_2, 29940_ = 5.53, *p* = 0.004), with higher pupil response to old (*z* = 3.23, *β* = 0.02, *p*_holm-bonferroni_ = 0.004) and CL (*z* = 2.37, *β* = 0.01, *p*_holm-bonferroni_ = 0.036) stimuli than new ones but no difference between old and CL stimuli (*z* < 1; Fig. 2a). This difference did not vary across the recognition period as revealed by the non-significant stimulus type by time interaction (*F*_18, 29926_ = 1.06, *p* = 0.39). The main effect of time was also significant (*F*_9, 29926_ = 51.48, *p* < 0.001) simply showing a significant linear increase in pupil response across time (*β*_linear_ = 0.11, SE = 0.005, *p* < 0.001). There was also a significant difference in pupil response across memory type (*F*_2, 29951_ = 24.02, *p* < 0.001; Fig. 2b), and a significant memory type by time interaction (*F*_18, 29926_ = 10.08, *p* < 0.001; see Fig. 3a–c) indicating differential pupil response during the recognition period (time) for the different memory outcomes, accompanied by a significant linear increase in pupil response across N, F, R (i.e., N < F < R; *β*_linear_ = 0.02, SE = 0.003, *p* < 0.001).

Importantly, the stimulus type × memory type (*F*_4, 29948_ = 3.83, *p* = 0.004) and the stimulus type × memory type × time (*F*_36, 29926_ = 1.81, *p* = 0.002) interactions were also significant indicating differential pupil response during the recognition period for F, R, N responses across old, new and CL stimuli. For new responses, as shown in Fig. 3a, similar pupil amplitudes were recorded at recognition across old, CL and new stimuli (all *p*s > 0.05). Familiar responses were accompanied by increased pupil amplitude for CL relative to old (*β* = -0.05) and new stimuli (CL > old > new; all *β* > 0.05; all *p* < 0.05) for timepoints 3, 5 and 6 (Fig. 3b). Finally, recollected CL and new stimuli produced similar pupil responses that significantly differed from old recollected stimuli earlier on (from timepoints 3 to 5; all *β* > 0.05 and all *p* < 0.05; Fig. 3c). After the 5th timepoint an increase in the pupil amplitude for CL stimuli was observed resulting in a significant linear effect with CL > old > new from the 8th to the 10th timebin (8th: *β*_linear_ = 0.05, SE = 0.02, *p* = 0.02; 9th: *β*_linear_ = 0.07, SE = 0.02, *p* = 0.003; 10th: *β*_linear_ = 0.10, SE = 0.02, *p* < 0.001). Taken together, these findings indicate sensitivity of the pupil response to true and false memories, either based on familiarity or recollection, but at different timepoints during the recognition period.

#### Experiment 2

**Figure 4:**
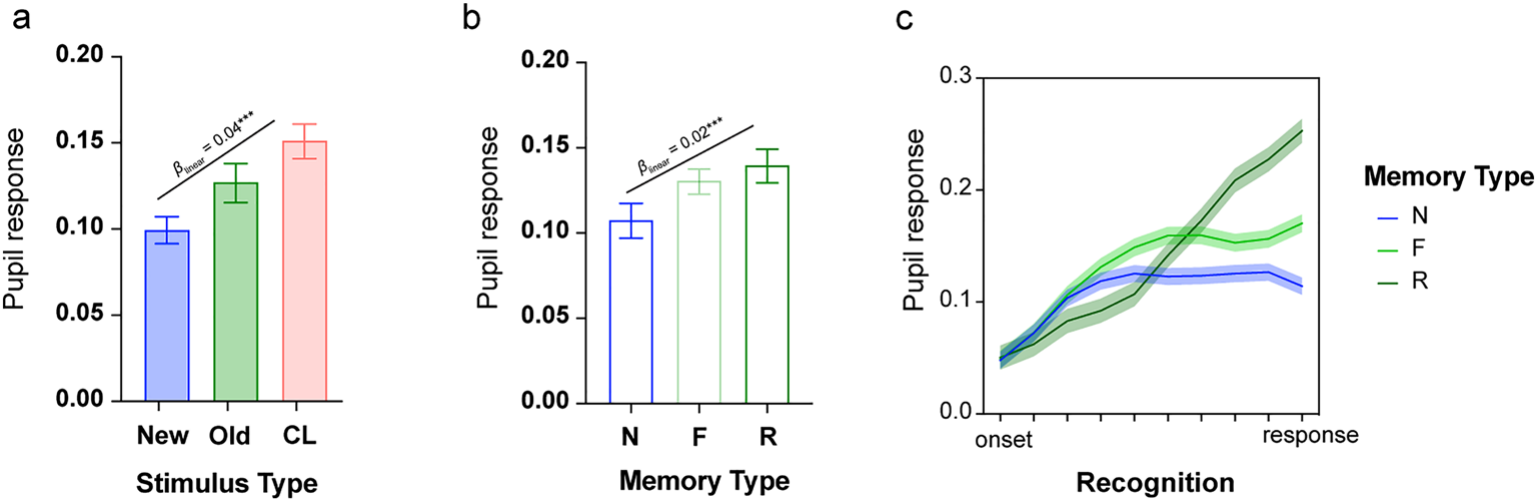
Pupil response across the different stimulus types (new, old and CL; a) and across reported memory outcomes (N = new, F = familiar, R = recollected; b and c) in Experiment 2. Error bars (**a**, **b**) and shaded areas (**c**) show the standard error of the mean. *** *p* < 0.001.

In Experiment 2, the analysis was conducted on a total of 3840 trials across participants, separated as 841 recollections, 1222 familiarity and 1774 new responses across old, new and CL stimuli. The linear mixed effects analysis on pupil response with fixed factors, stimulus type (old, new, CL), reported memory type (F, R, N) and time (10 time-bins) and the random effect of participants (intercept), showed differential pupil responses across stimulus types (*F*_2, 35526_ = 73.85, *p* < 0.001). This stemmed from a linear increase of pupil response across new < old < CL stimuli (*β*_linear_ = 0.04, SE = 0.01, *p* < 0.001; Fig. 4a). This difference did not vary across the recognition period as revealed by the non-significant stimulus type by time interaction (*F*_18, 35522_ = 1.59, *p* = 0.054). The main effect of time was also significant (*F*_9, 35522_ = 82.45, *p* < 0.001) simply showing a significant linear increase in pupil response across time (*β*_linear_ = 0.13, SE = 0.005, *p* < 0.001). There was also a significant difference in pupil response across memory types (*F*_2, 35348_ = 41.07, *p* < 0.001), and a significant memory type by time interaction (*F*_18, 35522_ = 11.83, *p* < 0.001) indicating differential pupil response across memory types with a significant linear increase in pupil response across N, F, R (i.e., N < F < R; *β*_linear_ = 0.02, SE = 0.003, *p* < 0.001; Fig. 4).

**Figure 5:**
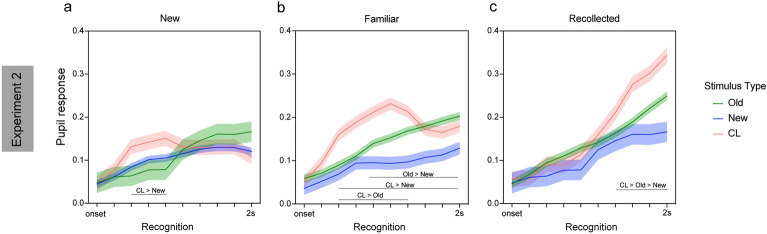
Pupil response timeseries at recognition for the different stimuli (old, new, CL) and reported memory – (**a**) new; (**b**) familiar (**c**) recollected in Experiment 2. Shaded areas show the standard error of the mean. Lines indicate significant effects at *p* < 0.05.

Importantly, the stimulus type × memory type (*F*_4, 35015_ = 16.68, *p* < 0.001) and the stimulus type × memory type × time (*F*_36, 35522_ = 3.70, *p* < 0.001) interactions were also significant indicating differential pupil response during the recognition period for F, R, N responses across old, new and CL stimuli. Specifically, for new responses (Fig. 5a) an increase in pupil amplitude for CL relative to new stimuli was found early on in the recognition period, from the 3^rd^ to the 5th timepoint (all *β*s > 0.042 and all *p*s < 0.022). For familiarity responses (Fig. 5b), pupil amplitude significantly increased for CL relative to old stimuli from 3^rd^ to the 7th timepoint (all *β*s > 0.04 and all *p*s < 0.007) and relative to new stimuli from the 3^rd^ timepoint to the end of the recognition period (all *β*s > 0.05 and all *p*s < 0.01). Also, for familiarity responses, the pupil amplitude for old stimuli significantly increased relative to new stimuli half-way through the recognition period (5th timepoint) until the end (all *β*s > 0.04 and all *p*s < 0.009). Finally, for recollected stimuli (Fig. 5c), after the 7th timepoint a rapid increase in the pupil amplitude for CL stimuli was observed accompanied by significant linear effect with CL > old > new (7th: *β*_linear_ = 0.046, SE = 0.021, *p* = 0.027; 8th: *β*_linear_ = 0.08, SE = 0.021, *p* < 0.001; 9th: *β*_linear_ = 0.10, SE = 0.021, *p* < 0.001; 10th: *β*_linear_ = 0.12, SE = 0.021, *p* < 0.001). Taken together, the findings from Experiment 2 are comparable to those from Experiment 1, indicating differential sensitivity of the pupil response to true and false memories, but discriminating, at the same time, the type of memory involved (familiarity versus recollection).

## Discussion

### Summary of findings

Pupil response has emerged as an important measure of cognitive processing and long-term memory retrieval^5–8,12^. In the present experiment we set out to investigate whether pupil response discriminates true from false memories. We reasoned that the capacity of the pupil response to discriminate true from false memories, will provide insights as to whether changes in pupil response constitute reflections of successful memory retrieval. The main findings can be summarised as follows: Firstly, the findings indicate that the pupil timeseries can discriminate true from false memories in terms of differential amplitude at different timepoints during the recognition period (temporal components). Secondly, the pattern of discrimination between true and false memories appears to be similar between visually and auditory presented stimuli, although the discrimination appears to be slightly more robust in the auditory condition, which coincides with more robust false memory effects in the same condition. Finally, the basis of recognition, either familiarity- or recollection-based, appears to result in different pupil dilation patterns not only for true memories, but more importantly for the purpose of the present study, for false memories too. Specifically, familiarity-based false memories were characterised by increased pupil responses, as compared to familiarity-based true memories, at an earlier temporal component during the recognition period. In contrast, recollection-based false memories were characterised by increased pupil amplitude as compared to recollection-based true memories at a later temporal component, closer to the end of the recognition period. The findings and their implications for pupillometry, memory theories and potential mechanisms involved in the generation of false memories are further discussed below.

### Pupil patterns discriminate true from false memories

Taking into account the sensitivity of the pupil response in memory formation and retrieval^5–8,12,13^, and previous indirect evidence for its capacity to discriminate veridical memories from false alarms^12^, we proposed three hypotheses related to the ability of the pupil response to discriminate true from false memories (see Introduction). The results from Experiments 1 and 2, consistently agree with the proposal that the pupil response at retrieval is modulated by both objective and subjective elements of memory retrieval and therefore both true and false memories were reflected on the pupil amplitude.

Specifically, it was found that the pupil response during the recognition period discriminated new, familiar and recollected stimuli. The general shape of this effect resembles what has been previously reported in the literature^5,13^, with a linear increase in pupil dilation from new to familiar and recollected stimuli (new < familiar < recollected). The observed differences in pupil response between familiar and recollected stimuli demonstrates the deployment of different processing strategies and cognitive mechanisms^5,6^. For example, a previous fMRI study^5^ suggested, the involvement of distinct brain networks for familiarity and recollection, whilst eye tracking data with the same paradigm demonstrated distinct pupil responses. The involvement of distinct neural pathways in familiarity and recollection have been consistently replicated in subsequent studies e.g.,^42,43^. It is therefore very likely that the mechanisms involved in the generation of false memories for familiar and recollected stimuli are partly distinguishable, as implied by the pupil response patterns found here.

Importantly, in the present study this old/new effect, was modulated by the veridical memory status of stimuli, critically discriminating between truly old from CL stimuli (see Figs. 2 and 4). For the stimuli reported as familiar, this took the form of an early pupil dilation increase for CL stimuli relative to old and new stimuli. On the other hand, for recollected stimuli, both experiments showed a consistent pattern of increased pupil dilation for CL stimuli, relative to old and new ones, at a later temporal component. Therefore, these findings indicate the pupil response can discriminate true from false memories, but the pupil patterns, and especially the timing of their differential amplitude, vary depending on the memory type that enables recognition.

As reported in the Introduction, only indirect evidence is currently available regarding the ability of pupil response to distinguish true from false memories. Studies using recognition memory paradigms without a false memory manipulation have provided mixed findings in relation to this question^12,19,22^. At the same time, the capacity of the pupil response to reflect memory encoding and retrieval is debated, with some researchers linking the pupil output to other factors, such as working memory load, arousal or response preparation^23–26^. While other accounts link encoding- or retrieval-related changes in pupil response directly to the engagement of memory networks and their differential neurotransmission under different memory conditions^8,18,50^ see also^51^. The present findings provide direct evidence for the ability of the pupil response to discriminate true from false memories making it a reliable indicator of retrieval success. Furthermore, examining the dynamic change in pupil response across time suggests that the mechanisms involved in familiarity-based and recollection-based false memories are dissociable. The nature of these mechanisms cannot be uncovered based on the pupil signal alone. However, below we present a proposal based on the temporal differences of the pupil signal identified in this study, known mechanisms critical for familiarity and recollection, and previously proposed mechanisms involved in false memory generation.

### Possible implications for false memory mechanisms

What can the differential temporal components identified in the present study tell us about the mechanisms involved in false memory generation? As noted above, pupil response patterns do not allow a direct answer to this question, but proposals can be formulated taking into account the timing of the pupil effects and the type of memory involved in each case. In general, fluency is considered a critical process contributing to feelings of familiarity^52–55^. We speculate here that increased pupil dilation related to false familiarity, at earlier stages of processing, reflects enhanced fluency due to spreading activation to critical lures at encoding or gist-consistency with encoded events. This proposal is consistent with the way spreading activation^4,32^ and fuzzy-trace prespectives^33^ explain the generation of false memories. Specifically, within a list of words at encoding, each word activates a unique trace, but it also spreads the activation to semantic associates, in most cases the CL word. Similarly, from a fuzzy-trace theory perspective, gist extraction from each word at study, makes it more likely for the respective CL word to be processed as an encoded event. According to these perspectives, CL words are fluent due to the summative activation during each word in a list. The enhanced fluency due to multiple instances of activation and gist enhancement, may lead to elevated pupil dilation relative to truly familiar stimuli. This fluency is more likely to be attributed to familiarity in the context of a recognition memory task^56^. The timing of this effect is also consistent with the well-documented fast and automatic nature of familiarity memory^57^.

On the other hand, source attribution and monitoring processes are key stages of recollection-related processing. Recovery of source and contextual associates and monitoring the validity of source attribution are critical for successful^58,59^ and unsuccessful^29,60^recall and recollection. Therefore, we propose here that in the case of recollection-based false memories the failing mechanism relates more to the later stages of processing, which may indicate erroneous source attribution and monitoring processes. These processes are computationally more intensive in the case of false recollections as presumably the ensuing memory search and source monitoring involve coming up with reasonable associative details, which may drive the elevated pupil dilation for false relative to true recollection.

### The effect of modality of presentation on false memory generation

Modality of presentation emerged as an important factor related to the rate of false memory at retrieval. Indeed previous studies have shown that visual presentation at encoding reduces false memory reports in recall and recognition tasks relative to auditory presentation^61–65^. One explanation previously proposed for this effect is that visual distinctiveness assists in discriminating studied items from critical lures, as the former have richer visual detail than non-presented similar items. Nevertheless, this heuristic cannot be easily applied in the case of auditorily studied stimuli, making them more prone to false memory generation at retrieval^66^.

Consentient with these previous observations, in the present study, false memory effects to CL items were more pronounced in Experiment 2 (auditory) relative to Experiment 1 (visual). The identified pupil response patterns were similar in the two experiments with two notable exemptions. Firstly, in Experiment 2, new responses to CL stimuli were accompanied by increased pupil dilation relative to correct rejections (new responses to new stimuli) at an early temporal component. Secondly, the amplitude differences between true and false memories were enhanced in Experiment 2. These findings support the previously proposed stronger false memory effects for auditory stimuli and are generally consistent with the visual distinctiveness explanation presented above. One difference between the two experiments was that recognition in Experiment 1 (visual) was self-paced, while in Experiment 2 (auditory) each trial lasted for 2 s. It is not known whether this difference, due to the requirement of delivering visual versus auditory stimuli, may have affected the behavioural and/or pupillary effects. Despite this difference, both experiments generated false memories and produced similar pupillary response patterns.

## Conclusion

Overall, this study shows that the pupil response can reliably discriminate between true and false memories. The temporal patterns of the pupil components suggest that the mechanisms involved in the generation of false memories differ depending on the type of memory supporting recognition decisions. We proposed that the pupil patterns reflect the different roles of fluency and source attribution and monitoring processes in generating familiarity-based versus recollection-based false memories. This proposal is consistent with the type and timing of pupil dilation effects and the nature of the established processes that drive different memory experiences, but warrants further investigation. Finally, replication using autobiographical memory paradigms could provide important insights into the generalisability of these findings.

### Supplementary Information

Supplementary Tables.

## Data Availability

Data/material and analyses outputs are available on: https://osf.io/sy8tj/?view_only=b1bfdc1a6c744c0499935bd2c1ef39cd.
