# Supplementary material for: Pupil response patterns distinguish true from false memories
Source: Sci Rep. 2023 Oct 11;13:17244. doi: 10.1038/s41598-023-44362-6 (PMC10567773; doi:10.1038/s41598-023-44362-6)
Supplement: Supplementary file 1 — Supplementary Tables. [file 41598_2023_44362_MOESM1_ESM.docx]

**Supplemental Material**

***Pupil response patterns distinguish true from false memories***

by Alex Kafkas, Travorn Brown, Nifemi Olusola, and Chaodong Guo

**Table S1**

**Proportion of familiar, recollected and new responses and RTs (in ms) across three types of stimuli at recognition for Experiment 1 and 2**

|  | Word type | | | | | |
| --- | --- | --- | --- | --- | --- | --- |
|  | Old | | New | | CL | |
| Experiment 1 | | | | | | |
| Response | Proportions | RTs | Proportions | RTs | Proportions | RTs |
| Familiar | 0.32 (0.12) | 1608.26 (414.69) | 0.22 (0.15) | 1606.91 (547.70) | 0.31 (0.17) | 1669.47 (516.15) |
| Recollected | 0.30 (0.17) | 1360.28 (285.77) | 0.12 (0.17) | 1571.39 (507.70) | 0.14 (0.16) | 1353.88 (345.09) |
| New | 0.37 (0.16) | 1412.91 (341.24) | 0.59 (0.25) | 1344.31 (339.51) | 0.42 (0.25) | 1509.29 (352.76) |
| Experiment 2 | | | | | | |
| Response | Old | | New | | CL | |
| Familiar | 0.36 (0.11) | | 0.21 (0.12) | | 0.43 (0.12) | |
| Recollected | 0.30 (0.15) | | 0.07 (0.10) | | 0.27 (0.14) | |
| New | 0.34 (0.14) | | 0.72 (0.19) | | 0.30 (0.10) | |

*Note:* Numbers in the parentheses are standard deviations.

**Table S2**

**Mixed models summary for the analysis of pupil response, response proportions and RTs. Reported memory type (F, R, N), type of stimulus (old, new and CL) and time (10 timebins; only for pupil response) were specified as the fixed factors with participants as the random effect**

| Model (using *lme4* call formatting in *R*) | R^2^_marginal_ | R^2^_conditional_ | AIC | ICC | Random Effect LRT |
| --- | --- | --- | --- | --- | --- |
| *Experiment 1* |  |  |  |  |  |
| 1) pupil ~ 1 + stimulus type + memory type + time + stimulus type:memory type + stimulus type:time + memory type:time + stimulus type:memory type:time+ (1 \| subjects) | 0.03 | 0.13 | 424.34 | 0.11 | *χ*^2^(1) = 3433, *p* < 0.001 |
| 2) proportions ~ 1 + stimulus type + memory type + stimulus type:memory type + (1 \| subjects) | 0.37 | 0.37 | -192.11 | 5.14x10^-17^ | *χ*^2^(1) < 1, *p* = 1.00 |
| 3) RTs ~ 1 + stimulus type + memory type + stimulus type:memory type + (1 \| subjects) | 0.08 | 0.59 | 4735 | 0.56 | *χ*^2^(1) = 172, *p* < 0.001 |
| *Experiment 2* |  |  |  |  |  |
| 1) pupil ~ 1 + stimulus type + memory type + time + stimulus type:memory type + stimulus type:time + memory type:time + stimulus type:memory type:time+ (1 \| subjects) | 0.05 | 0.06 | -4049 | 0.007 | *χ*^2^(1) = 164, *p* < 0.001 |
| 2) proportions ~ 1 + stimulus type + memory type + stimulus type:memory type + (1 \| subjects) | 0.61 | 0.61 | -416 | 0.00 | *χ*^2^(1) < 1, *p* = 1.00 |

*Note:* AIC = Akaike information criterion; ICC = intraclass correlation coefficient; LRT = likelihood ratio test. R^2^_marginal_ indicates the variance explained by the fixed effects relative to the total variance in the dependent measure, while R^2^_conditional_ indicates the variance explained by the fixed and random effects together relative to the variance in the dependent measure.
